# Mapping the global health employment market: an analysis of global health jobs

**DOI:** 10.1186/s12889-018-5195-1

**Published:** 2018-02-27

**Authors:** Jessica M. Keralis, Brianne L. Riggin-Pathak, Theresa Majeski, Bogdan A. Pathak, Janine Foggia, Kathleen M. Cullinen, Abbhirami Rajagopal, Heidi S. West

**Affiliations:** 1 Cadence Group, 1095 Zonolite Rd NE, Suite 105, Atlanta, GA 30306 USA; 2 Qual and Quant Analytics, Albuquerque, NM 87111 USA; 3 International Health Section, American Public Health Association, 800 I St NW, Washington, DC, 20001 USA; 4 François-Xavier Bagnoud Center, School of Nursing, Rutgers University, 65 Bergen Street, Newark, NJ 07101 USA; 5 Global Environmental Health LAB, Brooklyn, NY 11206 USA

**Keywords:** Employment, Employment market, Job market, Global health jobs

## Abstract

**Background:**

The number of university global health training programs has grown in recent years. However, there is little research on the needs of the global health profession. We therefore set out to characterize the global health employment market by analyzing global health job vacancies.

**Methods:**

We collected data from advertised, paid positions posted to web-based job boards, email listservs, and global health organization websites from November 2015 to May 2016. Data on requirements for education, language proficiency, technical expertise, physical location, and experience level were analyzed for all vacancies. Descriptive statistics were calculated for the aforementioned job characteristics. Associations between technical specialty area and requirements for non-English language proficiency and overseas experience were calculated using Chi-square statistics. A qualitative thematic analysis was performed on a subset of vacancies.

**Results:**

We analyzed the data from 1007 global health job vacancies from 127 employers. Among private and non-profit sector vacancies, 40% (*n* = 354) were for technical or subject matter experts, 20% (*n* = 177) for program directors, and 16% (*n* = 139) for managers, compared to 9.8% (*n* = 87) for entry-level and 13.6% (*n* = 120) for mid-level positions. The most common technical focus area was program or project management, followed by HIV/AIDS and quantitative analysis. Thematic analysis demonstrated a common emphasis on program operations, relations, design and planning, communication, and management.

**Conclusions:**

Our analysis shows a demand for candidates with several years of experience with global health programs, particularly program managers/directors and technical experts, with very few entry-level positions accessible to recent graduates of global health training programs. It is unlikely that global health training programs equip graduates to be competitive for the majority of positions that are currently available in this field.

## Background

The field that has now come to be known as global health has evolved substantially over the last fifty years. Driven by global pandemics such as HIV, increased foreign aid budgets from the U.S. and other high-income nations, the emergence of new multilateral institutions and NGOs such as the Gates Foundation, and increasing prioritization of country ownership of health programs, both the politics and the funding structure of global health work have shifted [1]. Global health has also experienced increased levels of attention and funding [2, 3]. Interest among students in high-income countries has increased as well, as evidenced by the impressive growth in the number of global health graduate programs [4, 5].

However, the influence of such changes on the global health job market has not been well studied, which has raised concerns about a potential mismatch between supply and demand [5]. Eichbaum et al. [4] initially explored this question with a pilot study of web-based job postings for global health employment opportunities; however, this provided only a “snapshot” of the current employment landscape, analyzing 178 job vacancies for positions based in low- and middle-income countries collected during two short periods, first in August 2013 and then again from November 2013 to January 2014. In order to gain a clearer understanding of the demands of the global health profession, and whether current training programs are adequately preparing students to enter the field, a more complete picture is needed of the current employment market. This includes the levels of experience, types of subject matter expertise, language and technical skills, and geographic experience required for available positions. To this end, we collected data on publicly posted global health jobs available to candidates authorized to work in the U.S. in order to characterize the skills and experience required for job applicants to be competitive for these positions.

## Methods

The aim of this study was to provide a descriptive analysis of a large sample of job vacancies in the current global health job market captured over a six-month period.

### Study design

In this cross-sectional study, data was collected from global health job vacancies posted to web-based, open (non-subscription) job boards, employment email listservs for global health and development, and global health organizations and consulting firms over a six-month period from November 2015 to May 2016. Vacancy descriptions were analyzed to characterize the most common types, levels, and required skills and experience of positions in the global health job market.

### Data collection

Job samples included in the master data set (S_1_) consisted of one posted job vacancy. A global health vacancy was defined as one soliciting applications for a paid non-internship position working with a project, program, or consultancy focused on a goal directly addressing human health. Positions with development programs not focused on health (e.g., agriculture, animal husbandry, microfinance, democracy/governance, and security), as well as positions only open to nationals from a particular country, were excluded. Both full- and part-time positions were included. Vacancies were gathered from twelve internet job boards, including Devex, DevNetJobs, Emory Public Health Employment Connection, Global Health Council, Idealist, Indeed.com (both the global health and international health job boards), Johns Hopkins Bloomberg JHSPHConnect, Peace and Collaborative Development Network, Public Health Institute newsletter, ReliefWeb, and USAJobs. Vacancies were also collected directly from the websites of 20 global health agencies and consulting firms, including Abt Associates, Camris International, CARE, Chemonics, Clinton Health Access Initiative, FHI 360, Gates Foundation, International Medical Corps, International Red Cross/Red Crescent, International Rescue Committee, John Snow International, Management Sciences for Health, Médecins Sans Frontières, PHI Global Health Fellows Program, Population Services International, RTI International, Samaritan’s Purse, Save the Children, Task Force for Global Health, U.S. Office of Foreign Disaster Assistance, World Health Organization, and World Vision. These job boards and employers were identified based on the authors’ collective knowledge of the global health industry and our 30 years of combined experience in global health job seeking. We collected 1254 total vacancy descriptions. Of these, 47 internships, unpaid (volunteer) positions, or positions for work not related to health were excluded. Of the remaining 1207 vacancies, 200 duplicate positions were eliminated, resulting in 1007 unique global health vacancies.

The data set for quantitative analysis (S_2_) was created by entering information on select characteristics of S_1_ into a spreadsheet. S_2_ data taken directly from each vacancy description included employer, position title, physical location of position, opening and closing dates (if applicable), education level required and preferred (for private- and nonprofit-sector vacancies), minimum number of years of overall professional experience and directly related experience required (for private- and nonprofit-sector vacancies), lowest and highest GS level (for US federal government vacancies), whether the position is term or permanent (for U.S. federal government vacancies), country or region of focus, whether proficiency in a second (non-English) language was required or preferred, up to two non-English languages desired, and whether overseas experience was required or preferred. For each vacancy, the two predominant areas of technical expertise required for the position were identified. After de-duplication, a unique alphanumeric ID was assigned, and private- and non-profit vacancies were classified by level as practicing clinician (clinical care or training responsibilities specified in vacancy announcement), entry-level (no more than three years of experience and no specialized technical knowledge or experience required), mid-level (two to five years of experience with some specialized technical knowledge or experience required), managerial (“manager” in position title and/or at least three years of project/program management experience required, plus financial, training, strategic planning, operations, or personnel management responsibilities specified in the vacancy announcement), technical/subject matter expert (at least two years of specialized experience required, plus consultative or training responsibilities, or management of technical aspects of projects/programs, specified in the announcement) or directorial (“director,” “chief of party,” “country representative,” or “president” in position title) according to job title, description, and the minimum number of years of experience required.

A qualitative data set (S_3_), consisting of samples with the text of each vacancy description (S_1_), was generated by simple randomized sampling of S_1_ without replacement, based on previously published sampling designs for mixed methods analyses [6–8]. To ensure an unbiased data set and sufficient sample size, 20% of the master dataset was used as the qualitative sample subset of S_1_. This had the effect of sampling from all six months and including samples from all sectors, which avoided both time- and sector-based biases that could have been introduced by a smaller subset.

### Statistical analysis

S_2_ data was imported and descriptive analysis performed using SAS 9.4 (SAS Institute, Cary NC). All postings within the master data set were included in the analysis. Job and employer/agency counts, job levels, requirements and preferences for education level, non-English language proficiency and overseas experience requirements, geographic focus, top technical expertise areas, and physical location were calculated as frequencies and proportions. For each technical specialty area, vacancies with a focus in that area were compared to the remaining vacancies in the dataset that did not have that technical specialty as a focus of the job duties in order to determine whether those positions were more or less likely to require non-English language proficiency and overseas experience. The comparisons were assessed by calculating Chi-square statistics.

### Thematic analysis

S_3_ data were imported into Dedoose Version 7.6.13 (SocioCultural Research Consultants LLC, Los Angeles, CA, USA) and analyzed using a thematic analysis approach. Content of the vacancies was classified either as a responsibility of the position or a desired qualification in the applicant. Position descriptions consisted of tasks, areas of responsibility, events, and deliverables that fell under the scope of the position as specified in the vacancy description. Candidate requirements included skill sets, experience with particular tasks or program areas, and individual character traits. A non-randomized sample of approximately 25% of the vacancies collected prior to February 2016 was used as a training and calibration set to develop a coding methodology and taxonomy for thematic analysis. This methodology was then used to analyze the S_3_ subset described above.

## Results

A total of 1007 global health job vacancies from 127 employers were collected for analysis. Of the 1007 total job vacancies, 88% (*n* = 884) were positions listed by 126 distinct employers in the private or non-profit sector. The remaining 12% (*n* = 123) were U.S. government positions within six different federal agencies.

### Private and non-profit sector vacancies

Among private and non-profit sector vacancies, there was a clear need for experienced global health professionals, including highly-trained technical specialists, program and project managers, and program directors (Table 1). Of the 884 vacancies, 40% (*n* = 354) were for technical or subject matter experts (median at least six years of related experience required), 20% (*n* = 177) for program directors or chiefs of party (median at least eight years of related experience required), and 16% (*n* = 139) were for program or project managers (median at least five years of related experience required), compared to 9.8% (*n* = 87) for entry-level and 13.6% (*n* = 120) for mid-level positions (Table 1). Additionally, 61% (*n* = 536) of vacancies specified a preference for candidates with a master’s degree, and an additional 9% (*n* = 82) specified a preference for applicants with a (non-clinical) doctoral degree.

**Table 1:** Private and non-profit global health job vacancies

		N	%
Total		884	100
Level	Entry	87	9.8%
	Mid	120	13.6%
	Manager	139	15.7%
	Technical/SME	354	40.0%
	Director/COP	177	20.0%
	Clinician	7	0.8%
Type of Degree Preferred^a^	Clinical (any)	87	9.8%
	Physician	68	7.7%
	Nursing	8	0.9%
	Bachelors	136	15.4%
	Masters (any)	536	60.6%
	MPH	61	6.9%
	Doctoral	82	9.3%
Non-English Language Proficiency	None	498	56.3%
	Preferred	226	25.6%
	Required	160	18.1%
Overseas Experience	None	500	56.6%
	Preferred	156	17.6%
	Required	228	25.8%
Geographic Focus^b^	None	426	48.2%
	West Africa	84	38.2%
	East Africa	76	
	Southern Africa	74	
	Central Africa	65	
	Sahel	14	
	Africa (general)	30	
	South Asia	32	6.9%
	Southeast Asia	26	
	Central Asia	8	
	East Asia	2	
	Asia (general)	4	
	Eastern Europe	6	0.7%
	MENA	32	3.6%
	LAC	33	3.7%

^a^Includes required degree if vacancy did not specify a preference for an education level beyond the minimum required. Degree totals will not add up to total number of vacancies, as some vacancies had multiple degree requirements

^b^Regional totals will not add up to total number of vacancies, as some vacancies had more than one regional focus

Over half (56.3%, *n* = 498) of vacancies did not have a non-English language proficiency requirement while the remainder either preferred (25.6%, *n* = 226) or required (18.1%, *n* = 160) at least one additional language (Table 1). Similar results were found regarding overseas experience, with 56.6% of vacancies containing no overseas work experience requirement, while 25.8% (*n* = 228) required and 17.6% (*n* = 156) preferred candidates who had worked outside of the U.S. Nearly half (48.2%, *n* = 426) of vacancies did not specify a country or regional focus. Of the remaining vacancies that did list a geographic focus, the most (38.2%, *n* = 338) listed at least one African country or region, while 6.9% (*n* = 61) specified work focused in Asia, 3.6% (*n* = 32) in the Middle East or North Africa, and 3.7% (*n* = 33) in Latin America or the Caribbean.

### U.S. federal government vacancies

Similar to the private and non-profit sector vacancies, the U.S. federal government vacancies were recruiting highly qualified candidates for mostly technical expert and program manager positions. Of the 123 U.S. government vacancies analyzed, 77% (*n* = 95) were for a General Schedule (GS)-level 13 or higher, and the median GS level for vacancies that adhered to the GS was 14 (Table 2). Additionally, 18.6% of vacancies were open for seven days or less, which may indicate that the hiring manager has an internal candidate in mind [9]. Approximately two-thirds (65.8%, *n* = 81) of vacancies were for permanent positions, while the remainder (33.3%, *n* = 41) were term positions (typically not to exceed two years).

**Table 2:** Global health job vacancies with U.S. federal agencies

		Agency				Total
		CDC	OFDA	State	USAID	
Total Vacancies		67	19	7	29	123
Days Vacancy Open	Median	14	28	13	13	14
	< 8 days (N)	15	–	2	4	23
Permanent or Term?	Permanent	53	2	5	21	81
	Term	14	17	2	6	41
Lowest GS Level	11	5	–	–	1	6
	12	5	3	–	2	10
	13	14	10	2	4	30
	14	14	5	2	14	35
	15	24	1	1	4	30
Median GS Level		14	13	14	14	14

### Location

Combined, global health job vacancies were divided almost evenly between being based in the U.S. (47%, *n* = 475) and overseas (53%, *n* = 532). However, most U.S. federal jobs (82.9%, *n* = 102) were based in the U.S., primarily in Atlanta (*n* = 60) and the Washington, DC area (*n* = 42), while more than half (57.8%, *n* = 511) of the private and non-profit sector jobs were located outside of the U.S. The most common regions for overseas position locations were in sub-Saharan Africa, followed by the Middle East and North Africa, South Asia, Latin America or the Caribbean, and Southeast Asia (Table 3). U.S.-based jobs in the private and non-profit job sectors were most commonly located in the Washington, D.C. area (*n* = 233) and Seattle (*n* = 52).

**Table 3:** Locations for private/non-profit sector and U.S. federal global health jobs

Region		PNP	USG	Total
North America	USA (DC Area)	233	42	275
	USA (Seattle)	52	0	52
	USA (Atlanta)	23	60	83
	USA (Other)	84	1	85
	Canada	3	0	3
Sub-Saharan Africa	Southern Africa	81	1	82
	Central Africa	89	1	90
	West Africa	79	1	80
	East Africa	70	2	72
	Sahel	15	1	16
MENA		39	1	40
Asia	South Asia	26	2	28
	Southeast Asia	20	2	22
	Central Asia	8	0	8
LAC		26	0	26
Europe		22	1	23

Regional totals will not add up to total number of vacancies, as some vacancies had more than one location option

### Technical focus area

Several common technical knowledge and skill categories emerged from the analysis of the job vacancies, as shown in Table 4. The most common skill area for both private/non-profit (34.5%, *n* = 305) and U.S. federal vacancies (45%, *n* = 58) was management of programs, projects, grants or portfolios, followed by HIV/AIDS (14.7% and 29.5% of private/non-profit and U.S. federal vacancies, respectively) and quantitative analysis and data management (8.9% and 12.4% of private/non-profit and U.S. federal vacancies, respectively). Other common technical focus areas in private and non-profit sector vacancies were monitoring and evaluation (14.3%, *n* = 126) and maternal and child health (8.4%, *n* = 74), while many U.S. federal vacancies were commonly focused on emergency response (14%, *n* = 18).

**Table 4:** Common technical knowledge and skill areas for global health job vacancies

Technical Focus Area	PNP Jobs		USG Jobs		Total Jobs	
	N	%	N	%	N	%
All	884	100%	123	100%	1007	100.0%
Management^a^	305	34.5%	58	45.0%	363	36.0%
HIV	130	14.7%	38	29.5%	168	16.7%
Monitoring & Evaluation	126	14.3%	3	2.3%	129	12.8%
Quantitative Analysis & Data Management^b^	79	8.9%	16	12.4%	95	9.4%
Maternal & Child Health	74	8.4%	1	0.8%	75	7.4%
Logistics, Operations, and Supply Chain Management	64	7.2%	0	0.0%	64	6.4%
Business^c^	55	6.2%	6	4.7%	61	6.1%
Soft Skills^d^	54	6.1%	3	2.3%	57	5.7%
Emergency Response	29	3.3%	18	14.0%	47	4.7%
Health Systems Strengthening	45	5.1%	0	0.0%	45	4.5%
Malaria	41	4.6%	3	2.3%	44	4.4%
Disease-Focused^e^	33	3.7%	8	6.2%	41	4.1%
Nutrition	37	4.2%	1	0.8%	38	3.8%
Sexual Health	36	4.1%	0	0.0%	36	3.6%
Water, Sanitation, & Hygiene (WASH)	34	3.8%	1	0.8%	35	3.5%
Tuberculosis	30	3.4%	5	3.9%	35	3.5%
Writing & Communications	28	3.2%	6	4.7%	34	3.4%
Pharmaceuticals	24	2.7%	0	0.0%	24	2.4%

Technical specialty areas will not add up to total number of vacancies, as most vacancies had more than one technical focus

^a^Management of programs, projects, grants, or portfolios

^b^Data analysis, surveillance, epidemiology, statistics, informatics, etc.

^c^Administration, coordination, coalition-building, partnership development

^d^Finance, business development, fundraising, HR, marketing

^e^NTDs, Ebola, Guinea worm, infectious disease, hepatitis, polio, etc

For private and non-profit sector vacancies, certain technical focus areas were significantly associated with requirements for non-English language proficiency and/or previous overseas work experience (Table 5). Jobs with a focus in maternal and child health and emergency response were both significantly more likely to require proficiency in a language other than English (*p* < 0.0001 and *p* < 0.0158, respectively) and overseas experience (*p* = 0.0016 and *p* = 0.0396, respectively). Jobs focused in HIV/AIDS (*p* = 0.0437) and sexual health (*p* = 0.0004) were more likely to require overseas experience. Vacancies with a focus on health systems strengthening were significantly less likely to require proficiency in a second language, and jobs in quantitative analysis and data management and those with a focus on non-technical “soft skills” (interpersonal and communication skills that enable professionals to interact effectively and work well with others) were less likely to require overseas experience.

**Table 5:** Second language and overseas experience global health job requirements by technical focus area

Technical Focus Area	PNP Jobs	Second language required or preferred			Overseas experience required or preferred		
	N	N	%	*p*-value	N	%	*p*-value
All	**884**	**386**	**43.7%**		**384**	**43.4%**	
Management^a^	305	140	45.9%	0.3305	144	47.2%	0.1004
Monitoring & Evaluation	126	58	46.0%	0.563	47	37.3%	0.1334
HIV/AIDS	130	49	37.7%	0.1371	67	51.5%	**0.0437**
Quantitative Analysis & Data Management^b^	79	32	40.5%	0.553	26	32.9%	**0.0479**
Maternal & Child Health	74	49	66.2%	**< 0.0001**	45	60.8%	**0.0016**
Logistics, Operations, and Supply Chain Management	64	27	42.2%	0.8045	28	43.8%	0.9584
Business^d^	55	21	38.2%	0.1142	18	32.7%	0.3273
Soft Skills^c^	54	18	33.3%	0.3972	20	37.0%	**0.0979**
Health Systems Strengthening	45	14	31.1%	**0.0813**	23	51.1%	0.2865
Malaria	41	19	46.3%	0.7235	23	56.1%	0.1663
Nutrition	37	19	51.4%	0.3355	16	43.2%	0.9804
Sexual Health	36	19	52.8%	0.2604	26	72.2%	**0.0004**
Water, Sanitation, & Hygiene (WASH)	34	12	35.3%	0.3156	12	35.3%	0.3285
Disease-Focused^e^	33	18	54.5%	0.199	17	51.5%	0.3401
Tuberculosis	30	13	43.3%	0.9703	13	43.3%	0.9905
Emergency Response	29	19	65.5%	**0.0158**	18	62.1%	**0.0396**

Technical specialty areas will not add up to total number of vacancies, as most vacancies had more than one technical focus

^a^Management of programs, projects, grants, or portfolios

^b^Data analysis, surveillance, epidemiology, statistics, informatics, etc

^c^Administration, coordination, coalition-building, partnership development

^d^Finance, business development, fundraising, HR, marketing

^e^NTDs, Ebola, Guinea worm, infectious disease, hepatitis, polio, etc

Bold values indicate that the difference between the percentage of job vacancies in the given category requiring or preferring proficiency in a second language or previous overseas experience, compared to the percentage of the entire sample, was statistically significant. The significance threshold was set at .05

### Qualitative analysis

The thematic analysis was performed by analyzing a subset of 203 vacancy descriptions (174 private/non-profit and 28 government). The categories under which job duties and responsibilities specified in vacancy descriptions were classified were program operations, relations, design and planning, communication, and management. Program operations predominantly consisted of implementation and oversight of activities, program support, and administrative duties. Relations included coordinating activities with partners, stakeholders, agencies, and other departments within the organization. Communication included both oral and written and encompassed interpersonal communications, presentations, representing the organization in meetings and at other events, and web-based communication such as social media. Management responsibilities included those related to personnel (e.g., hiring, training, and mentoring) as well as programmatic skills and operations (e.g., grants, knowledge, and information).

Candidates’ desired qualifications primarily fell into previous work experience and required skill sets. Predominant categories of desired previous work experience generally mirrored those found in position duties, with relations, management, and operations being some of the most common. The most common categories of skills were communication, software proficiency, management, and data analysis. Similar to communications responsibilities in listed job duties, vacancies typically specified a desire for candidates with strong verbal or written communications skills. However, management and analytical skills were often poorly defined. Among software proficiencies, Microsoft Office suite programs and statistical analysis packages (SPSS, STATA, and SAS) predominated.

## Discussion

Our analysis of global health vacancies with both the U.S. federal government and private and non-profit organizations shows a substantial need for candidates with several years of experience with global health programs, particularly program managers/directors and technical experts. Nearly two thirds of all private and non-profit sector vacancies were for technical experts or program directors. Similarly, the majority (81%) of U.S. federal government positions were listed at General Schedule (GS) 13 level or higher. The highest level on the General Schedule that an external applicant can generally qualify for on the basis of education is GS-11, [10] or a GS-12 for some research positions [11]. Positions with GS levels from 12 to 15 are considered “mid- to senior-level” positions (Ibid). According to the classification standards for the public health program specialist series, the GS-13 position classification for the Public Health Advisor job series states that the role “require[s] advanced occupational expertise” and “provide[s] technical and administrative guidance necessary to resolve matters which are very controversial, complicated, or set general precedent.” [12]. This experience level could be considered the equivalent of that required for a technical or subject matter expert level position with a private or non-profit employer. Over one third of private and non-profit positions, and nearly half of all U.S. government positions, called for demonstrated experience in program management. Need for technical expertise in HIV/AIDS, monitoring and evaluation, and data analysis and management also emerged.

The thematic analysis also revealed a tendency for position scope to include, and for candidates to possess experience with, a wide variety of high-level and often unrelated responsibilities, including (but not limited to) stakeholder engagement, project design, data analysis, grant writing, financial management, technical training, and fundraising. It was not uncommon for job vacancies to call for candidates with thorough knowledge and/or multiple years of experience in four or more of such areas, many of which are quite specialized. While it may be more common for global health professionals in resource-poor settings to “wear many hats,” it is unlikely that recent graduates of global health training programs would possess skills or experience of this breadth.

In addition to analyzing global health job vacancies, we identified 27 U.S.-based MPH programs with a global health concentration in 2016. We reached out to all of the programs to learn what year their global health MPH concentration was established and approximately how many students graduated with a global health MPH each year. We received responses from 13 of the programs we identified, which estimated that a total of 280–300 students graduate with an MPH in global health from those programs each year. These figures represent less than half of U.S. global health MPH programs. Our analysis identified only 87 entry-level global health positions over six months, suggesting that that the number of global health MPH graduates entering the job far outstrips the number of entry-level positions available in a given year. Our findings echoing concerns among global health programs that jobs for their graduates are limited, and that the supply of graduates is outpacing the demand [3].

A substantial amount of academic attention and commentary has been paid to the development and assessment of competencies in global health education [13]. Such competencies are typically developed through a combination of literature searches, reviews of existing competencies taught by global health programs, and outreach to global health academic and professional communities [13, 14]. This process results in training programs in high-income countries using frameworks built through consensus with other training programs in high-income countries, and through review of literature published predominantly by research institutions in high-income countries, [15, 16] rather than the host countries in which the graduates of such programs ostensibly seek to work. Additionally, Eichbaum has observed that global health programs may use their competencies to establish a unique identity to compete with similar programs in attracting students, which serves the program’s own interests rather than those of local health systems in which their graduates would seek employment [13, 17]. Eichbaum suggests [13] that the reason that global health trainees from institutions in high-income countries so often experience difficulty in host country settings is that the training they receive is built on educational philosophies and values of the high-income country in which their institution is based, rather than the host country of the setting they wish to serve. This may help to explain why so many the vacancies we analyzed called for applicants with multiple years of experience. For our entire data set, the median number of years of experience required was five. Working and relationship-building in the global health profession is highly dependent on local contexts, [13] and employers believe that these skills can only be gained through related experience, as they are not adequately developed in global health education programs.

Our analysis included several limitations. First, it is possible that many of the job vacancies captured by our analysis may not have actually been available to external candidates. Employers may intend to fill a vacancy with an identified recruit or internal candidate, but are required by laws, regulations, or organizational policies to post the vacancy [18]. This is also true for hiring managers in U.S. federal agencies, who are required to fill civil service positions through “fair and open competition” but frequently prefer to hire internal candidates or, in some cases, do not open positions to external candidates at all [19]. A 2011 survey conducted by the U.S. Merit Systems Protections Board found that, in cases where positions were not opened to external candidates, the top reason, cited by 51% of human resources staff, was that there were “plenty of highly-qualified internal candidates.” Additionally, 38% indicated that hiring managers often already had someone in mind, and 18% responded that the expertise could not be found in the private sector, indicating the belief that only candidates with previous federal government experience could perform the job duties [14]. In our sample, global health vacancies posted by federal agencies often had the same position posted in a separate vacancy open only to internal candidates, so it is possible that the hiring manager already had an intended or preferred candidate for the position. Additionally, several of the positions were posted for an anticipated need. If project funding was interrupted or not renewed, or the contract bid not accepted, those positions may have never been filled and thus might not be representative of the job market.

Conversely, some vacancies for open positions may not be publicly posted. Non-government jobs can be filled through networks or recruiting efforts so that hiring managers can avoid sifting through applications from unqualified candidates. It is also possible that we did not capture all positions available in the global health organizations we targeted, as these employers may have posted jobs on boards or in locations other than the sites which we had access to, such as subscription-restricted job boards or listservs. In addition, our data collection period only spanned six months from November to May and therefore may not have captured possible seasonal hiring increases in the summer or fall. Finally, our analysis captured relatively few clinical practice positions such as physicians, nurses, dieticians, dentists, optometrists, and mental health practitioners. While it is possible that many clinicians who provide services in the global health field do so on an unpaid basis, we may also have not identified enough sources of paid clinical practice vacancies.

## Conclusions

Overall, there were very few entry-level positions, those that might be accessible to recent graduates of a global public health program. Our findings echo concerns among global health programs that jobs for their graduates are limited, and that the supply of graduates is outpacing the demand [3]. Most employers in the global health field primarily hire technical experts and professionals with many years of experience in research or project implementation [2]. While the number of students graduating from global health training programs is increasing, the jobs most frequently sought typically require at least five years of field experience, making graduates unqualified to apply [5]. Furthermore, a recent survey of project directors at organizations implementing projects funded by the USAID Global Health Bureau found that two-thirds believed that MPH and global health programs did not adequately prepare students with non-clinical skills for the profession, [20] indicating that the training provided by global health graduate programs may not be well matched to the professional needs of the hiring market. This concern is underscored by the significant cost of U.S. global health MPH programs - a median of approximately $54,000 - which graduates will have to manage, in addition to any debt from their undergraduate programs. (This estimate is based on our identification of 27 MPH programs with a global health concentration in the fall of 2016. Median tuition was calculated based on graduate tuition for 60 credit hours.)

Global health training programs can help graduates prepare to work in global health by integrating an understanding of the global health career market into the curriculum and career assistance programs. Faculty and advisors should work with students to ensure realistic expectations of career prospects immediately upon graduation and provide concrete guidance on how to develop areas of technical expertise and cultivate a career for eventual entry into the global health field. Students should be coached on how to choose internships and practica that will provide experience that is valuable and directly applicable to their chosen career.

Universities that provide global health training programs have a responsibility not only to ensure that students and graduates are provided accurate information about career opportunities, but also to provide education and training that addresses unmet need in the global health field. Institutions should assess their curricula and training opportunities within the employment landscape. Additional research on the employment needs of the profession, such as key informant interviews with current global health practitioners, could strengthen the understanding of what is needed in the global health field and how aspiring global health professionals, and the programs that train them, can best meet the need to prepare a competitive global public health workforce and to help minimize a future workforce shortage.
